# Implementing Decentralized Clinical Trials in Australia through Teletrials: Where to From Here?

**DOI:** 10.1007/s43441-024-00658-x

**Published:** 2024-04-29

**Authors:** Tanya Symons, Anne Woollett, John Zalcberg, Lisa Eckstein

**Affiliations:** 1 T Symons Associates Pty Ltd, Sydney, NSW Australia; 2 TrialHub, Alfred Health, Melbourne, VIC Australia; 3 Cancer Research Program, School of Public Health, Monash University, Clayton, Australia; 4 Department of Medical Oncology, Alfred Health, Melbourne, Australia; 5 CTIQ and Bellberry Limited, Adelaide, Australia

**Keywords:** Teletrials, Satellite sites, Decentralized trials, Capacity building, Equity

## Abstract

Implementation of decentralized approaches can improve access to clinical trials. The Australian government has focused on a teletrial model, which resources and upskills health care organisations to enable collaboration in trials to extend to rural and remote areas. This commentary describes the Australian teletrial model, its context within the established DCT model, its value, and likely challenges moving forward.

## Introduction

Trial-related travel is perceived by many participants as inconvenient and burdensome [1, 2]. One survey indicated that 49% of respondents felt trial participation disrupted their daily routine [3]. 

The burden of travel disproportionately affects access to trials in rural and remote communities, presently accounting for 28% of the Australian population [4]. Trial sites tend to be opened in metropolitan areas, due to their proximity to teaching hospitals and universities, as well as access to a critical mass of clinician expertise. This likely contributes to the underrepresentation of rural and remote communities in clinical trials [5]. 

Decentralized approaches to trial conduct provide a possible solution to this disconnect [2]. A decentralized clinical trial (DCT) is a trial *where some or all the trial-related activities occur at locations other than centralised clinical trial sites* [6]. In 2021, the Australian Government invested heavily to support the implementation of DCTs. This investment has focused on the ‘teletrial model’, which differs from the established DCT model outlined in guidelines published by the Food and Drug Administration or the European Medicines Agency [7, 8]. 

This commentary describes the Australian teletrial model, its context within the established DCT model, its value, and likely challenges moving forward.

## The Teletrial Model

In 2015, the Clinical Oncology Society of Australia (COSA) piloted the Australasian Teletrial Model [9]. This evaluation led to further government funding for three programs to support the development of teletrial infrastructure (Table 1), the largest of which is the Australian Teletrial Program (ATP).

**Table 1:** Government Funding for Equitable Access to Clinical Trials

Award	Recipient	Planned Activity
$75.2 million	The Australian Teletrial Program (ATP) Department of Health Queensland	To establish Regional Clinical Trial Coordinating Centres (RCCCs) in QLD, WA, VIC, TAS, SA and the NT to establish infrastructure and to enrol more than 5000 patients into trials in RRR areas. The grant also supports the establishment of new RRR trial locations in each participating state/territory.
$30.6 million	The Rural, Regional and Remote (R3) clinical trial enabling program. NSW Ministry of Health and ACT Health	To progress infrastructure projects in RRR areas, including work to improve virtual trial capacity, increase trial awareness, improve trial recruitment and retention and professionalise trial services. Three clinical trial support units (CTSUs) support the delivery of trials in regional NSW.
$18.6 million	ReViTALISE Border Medical Oncology Research Unit (BMORU)	To progress several initiatives in areas of unmet need to provide equitable access to cancer trials in RRR Victoria, which focuses on providing access to high-quality trials to regional Victorians and supporting the development of rural infrastructure.

**Fig. 1:**
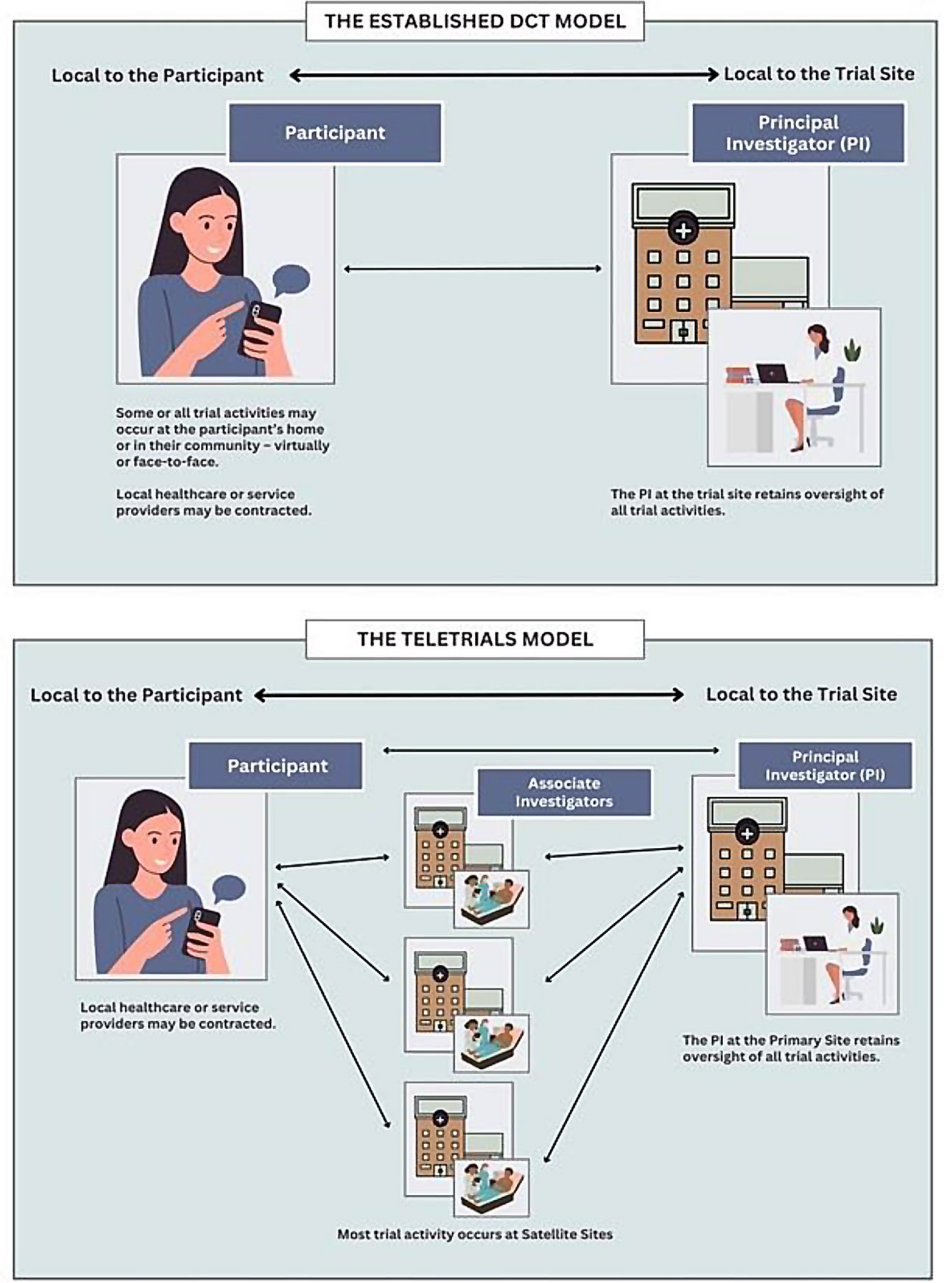
Teletrials versus established DCT model

Like other DCTs, teletrials are promoted as a means of improving the accessibility of trial sites. The key difference between the teletrial model and the established DCT model is that teletrials interpose additional, regionally dispersed trial sites between the Principal Investigator and trial participants (Fig. 1). The approach is operationalized through the establishment of *a cluster -* a hub and spoke model in which a ‘*Primary Site’* supervises one or more ‘*Satellite Sites’* located closer to participants’ homes.

**Fig. 2:**
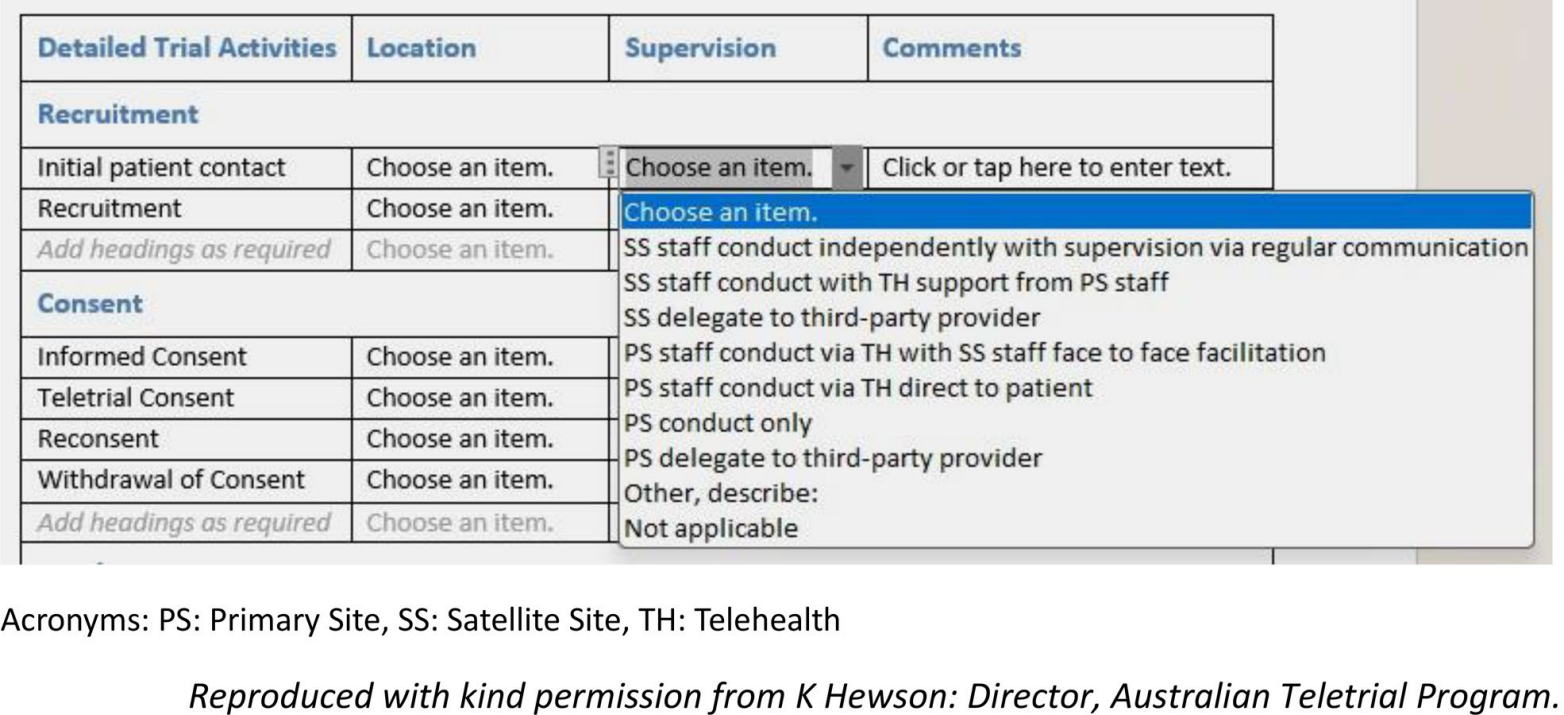
Risk-based trial oversight options outlined in the supervision plan developed by the Australian teletrial program (ATP)

In the teletrial model, Primary Sites take responsibility for all trial activity within *the cluster* and for communication with the sponsor. The Principal Investigator (PI) maintains oversight of trial activities carried out by Satellite Site staff (associate investigators, coordinators, pharmacists, etc.) [2]. Presently, a national supervision plan is being developed that enables risk-based oversight of activities conducted by the Satellite Site whereby the type and extent of oversight is influenced by factors such as the experience and capabilities of staff located at the Satellite Site and the complexity of the trial (Fig. 2).

Several other resources have been developed to support the implementation of teletrials in Australia.^1^ To date, the ATP has conducted 90 teletrials across 37 trial sites and aims to enroll 5,000 teletrial participants by 2026. An independent, mixed-method evaluation of the ATP Program will report in 2024 and 2026 [10]. 

Data collected to evaluate the programs include participant diversity and participant location, using the Modified Monash Model [11] to categorize remoteness. Participant experiences in teletrials and the cost effectiveness of the model are also evaluated [12, 13]. 

## Value of the Teletrial Model

Teletrials broaden the type of trial that can adopt a decentralised approach. As such, they offer opportunities to improve access to trials while maintaining a necessary level of trial protection (e.g. access to emergency care). In this way, treatments/interventions that require the level of clinical oversight and expertise available in a healthcare organization become feasible in a decentralized setting.

Teletrials may also improve access to trials that require continuity when trial assessments are performed [7]. For example, assessments performed by local healthcare providers may be less precise or more variable than assessments conducted at trial sites by dedicated trial staff [7]. Given the importance of data validity, in a teletrial these assessments can remain the responsibility of trained trial staff. Furthermore, the challenges associated with the new technologies used to carry out DCTs, including inadequate validation of novel digital tools or outcome measures, or inadequate medical oversight by investigators due to more complex data flow, will be less common in teletrials.

Additional advantages of the teletrial model include:

- *Building trial capacity and networked healthcare in rural and remote areas*: Ideally, as Satellite Sites gain critical trial infrastructure and experience through participation in teletrials, they will increase their capacity to act as Primary Sites in the future. The use of teletrials to mentor, upskill and supervise staff addresses a major systemic problem in rural and regional communities - the lack of trained and experienced investigators (and other qualified trial staff). This may also assist in the retention of skilled clinical staff outside major metropolitan centres [14]. Ongoing relationships between participating institutions may translate to future collaborative activities.
- *Improving equity of trial access to populations unwilling or unable to engage with technology*: Many DCT models rely on technology-centric implementation approaches. While broadly improving access, these trials may also entrench inequity by marginalizing those with poor internet access, poor digital literacy or those without suitable smart devices [15]. Teletrials maintain the human-centric model of most traditional clinical trial operations, which may be preferable for some participant groups.
- *Improving participant wellbeing*: For some participants, the face-to-face interaction built into the teletrial model may be preferable to more individualized DCT models; for example, by lessening feelings of isolation and promoting the relationships between participants and their treating team.
- *Reducing sponsor burden*: In a teletrial, the sponsor’s contractual relationship is generally with the Primary Site (although other models have been explored), which then subcontracts to Satellite Sites. Therefore, after the initial due diligence checks at the establishment of a new teletrial cluster, sponsors may benefit from increased and more diverse recruitment without a significant increase in the burden of opening and managing Satellite Sites. That said, compensating Satellite Sites for costs they incur may add complexity to the budgeting and contractual process.

## Challenges Associated with the Teletrial Model

While teletrials enhance the DCT landscape, they also present some challenges:

- *Implementation challenges*: The time and resources associated with opening Satellite sites has been reported as a limiting factor in the uptake of the teletrials model in Australia [2, 9]. Systemic issues within the health system—including workforce shortages and the lack of time and capacity for clinicians to conduct research—tend to be especially pronounced in rural and remote areas. As technology to support virtual health care develops, DCTs designed with these technologies will bypass at least some of these systemic issues.
- *Governance issues*: Researchers report complications and delays in the process of securing institutional (governance) approval for teletrials at both the Primary and Satellite Sites [16]. Although efforts are underway to address these issues [17], the challenges that researchers have identified more generally (non-transparency of requirements, variability in information required by different institutions) will be especially acute in the context of teletrials. This partly relates to the larger number of sites opened and the inexperience of many Satellite Sites in managing research governance processes. That said, investment in education, processes and templates could significantly mitigate this issue, and anecdotal reports suggests such a shift has already occurred in the state of Queensland.
- *Lack of flexibility in relationships and geographic regions*: The teletrial approach is based on hub-and-spoke clusters of Primary and Satellite Sites that may not accommodate the diverse and multifaceted relationships between research institutions that have arisen over time. Moreover, reliance on Satellite Sites may allow for less geographic reach compared to fully decentralised trials.
- *Lack of focus on other types of DCT*: Although the significant investment in capacity building will benefit all types of trial, the focus on the teletrial model, to the exclusion of other technology-driven DCTs, may lead to Australia being less prepared for these trials.

## Discussion

The ongoing success of the Australasian Teletrial Model will depend on its ability to address its current implementation challenges. Research governance processes, for example, will need to be streamlined and harmonized to ensure that Satellite Sites can be opened quickly. In addition, as the clinical trial sector in Australia benefits significantly from inbound investment from commercial sponsors [17], their willingness to use this model will be critical to its sustainability. Australia competes with many other Asia-Pacific countries for this investment, so sponsors must be confident that the model will be more efficient in the long run and ultimately value for money.

Although significant investment in capacity building and trial infrastructure will benefit all DCT, extending the teletrial programs to encompass a broader range of DCT models will further enhance Australia’s current position as an attractive destination for international sponsors.

Teletrials aim to facilitate clinical trials that enable rural and remote populations to take part in trials. As Satellite Sites mature, they become Primary Sites that in turn support the upskilling of new Satellite Sites.

## Conclusion

The significant investment in the teletrial model to build trial capacity in regional and remote areas demonstrates the Australian government’s commitment to clinical trials and its intention to promote more equitable access. Teletrials are a valuable DCT model as they increase the number of trials/trial activities that can be conducted closer to particpants’ homes. The next step will be to broaden the Australia’s investment in DCTs to include more technology driven models.

## Footnotes

1 For example, the Australian Teletrial Program (ATP), has published a comprehensive suite of resources (https://australianteletrialprogram.com.au/resources/).
